# Respiratory Arrest From Bilateral Hypoglossal Nerve Paralysis

**DOI:** 10.7759/cureus.98234

**Published:** 2025-12-01

**Authors:** Charlyn N Gomez, Sunny J Haft

**Affiliations:** 1 Department of Otorhinolaryngology - Head and Neck Surgery, University of Maryland School of Medicine, Baltimore, USA

**Keywords:** airway, bilateral hypoglossal nerve palsy, critical airway, emergency tracheostomy, hypoglossal nerve paralysis, necrotizing fasciitis (nf)

## Abstract

Bilateral hypoglossal nerve paralysis (BHNP) can cause airway compromise due to tongue collapse and impaired secretion management. This report underscores the importance of timely airway management. We present a case of a 52-year-old male with cervical necrotizing fasciitis of the left and right submandibular area who underwent multiple debridements, resulting in a BHNP. Sudden respiratory arrest occurred 12 hours after extubation, likely secondary to a combination of supine position, prolapse of a paralyzed tongue base, and poor secretion tolerance. An emergent bedside cricothyroidotomy was successfully performed. In this case, we emphasize the importance of early intervention for patients presenting with signs of BHNP, as well as careful airway surveillance following neck surgery.

## Introduction

Bilateral hypoglossal nerve paralysis (BHNP) is a rare event, occurring secondary to surgery, trauma, or intubation [1-13]. The hypoglossal nerves innervate nearly all intrinsic and extrinsic muscles of the tongue, making them essential for articulation, swallowing, and airway patency [14]. The sequelae of complete immobility of the tongue may include dyspnea, dysarthria, dysphagia, and the inability to tolerate oral secretions [5-8,10]. These complications not only impact quality of life but can be fatal if decompensation occurs due to airway compromise. Although respiratory decompensation is not a universal finding in isolated bilateral hypoglossal nerve palsy, it may occur when there is concurrent involvement of other lower cranial nerves [15] or when airway protection is compromised due to severe dysphagia and secretions. Given its rarity and wide range of etiologies, no consensus has been established on airway management in bilateral hypoglossal nerve palsy [5,16]. We present a rare case of BHNP secondary to necrotizing fasciitis to illustrate the potential for acute airway decompensation. Through a review of similar cases, we underscore the need for early recognition and proactive airway protection in this high-risk population. Accordingly, the objective of this case report is to illustrate how BHNP can precipitate abrupt airway compromise and to reinforce the importance of vigilant monitoring and early intervention.

## Case presentation

**Figure 1:**
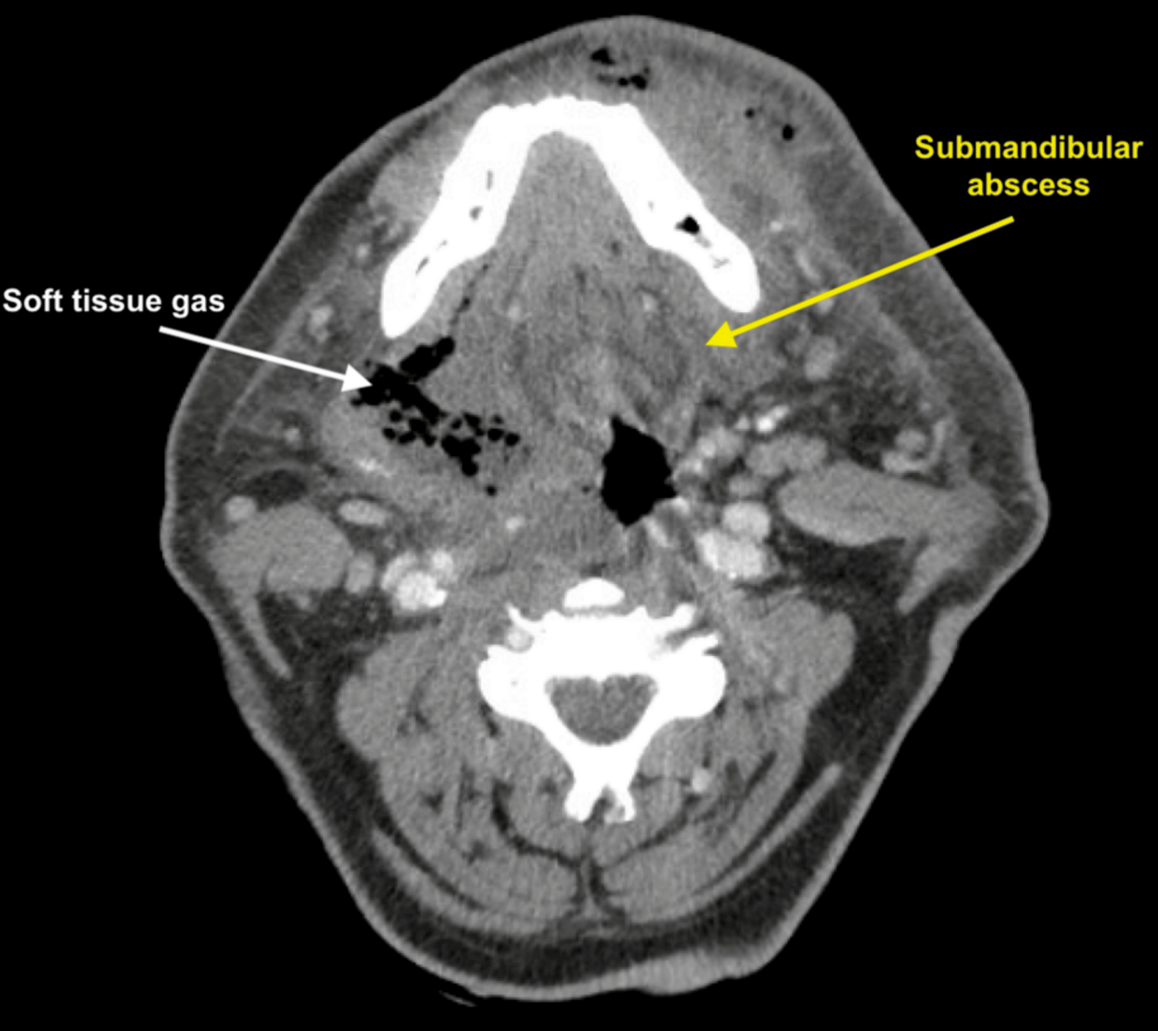
Pre-operative CT soft tissue neck An ill-defined collection of fluid and air, and associated skin thickening at the left anteromandible, along with a submandibular abscess (yellow arrow). There is extensive fat stranding seen in this region. Additionally, soft tissue subcutaneous air is seen within the right floor of the mouth and along the musculature of the floor of the mouth (white arrow).

A 52-year-old male with two days of progressive submandibular swelling, pain, and dysphagia was intubated at an outside hospital and transferred to our tertiary care institution with concern for necrotizing fasciitis. On arrival, he was tachycardic, febrile, and hypotensive. CT demonstrated a left submandibular abscess with extensive soft tissue gas in the floor of the mouth that extended to Level I of the contralateral neck, as shown in Figure 1. 

**Figure 2:**
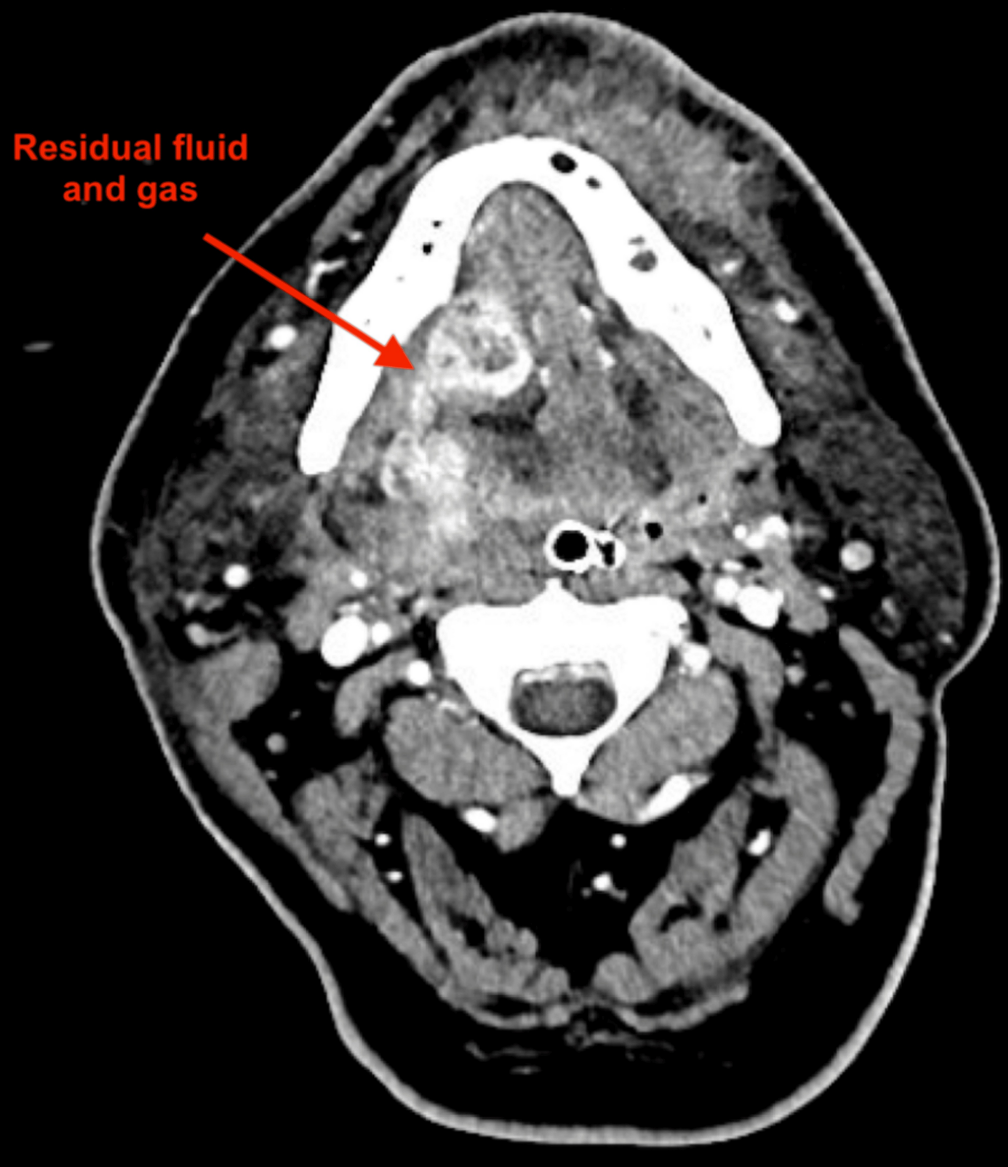
Post-operative CT soft tissue neck Interval surgical changes from drainage and debridement with residual gas and fluid collection (red arrow) medial to the right facial artery.

He was taken emergently to the operating room for transcervical debridement, where he was found to have extensive devitalized soft tissue and murky grey drainage throughout bilateral level I of the neck. Extensive debridement was performed until healthy bleeding tissue was encountered at all margins. Blunt dissection was used to expose areas of devitalized tissue along the left mandible and extending through the mylohyoid and geniohyoid muscles into the right floor of the mouth. Intraoperatively, it was found that teeth #19, 21, and 22 were necrotic and could have possibly contributed to the infection. Resultingly, OMFS and ENT coordinated the extractions of those teeth. Notably, the patient had an HgbA1c of 7% on admission, indicative of poor glycemic control and contributing to infection risk. Microbiology results included moderate growth of *Streptococcus anginosus* on neck abscess bacterial culture, as well as light growth of multi-drug-resistant *Pseudomonas aeruginosa* on submandibular tissue biopsy culture. There was no growth on two blood cultures. With the guidance of the infectious disease consultants, the patient was initially treated with ampicillin-sulbactam, vancomycin, and clindamycin irrigation through postoperative day four. He was then transitioned to piperacillin-tazobactam. Figure 2 demonstrates surgical changes from drainage and debridement, along with residual gas and fluid collection.

**Figure 3:**
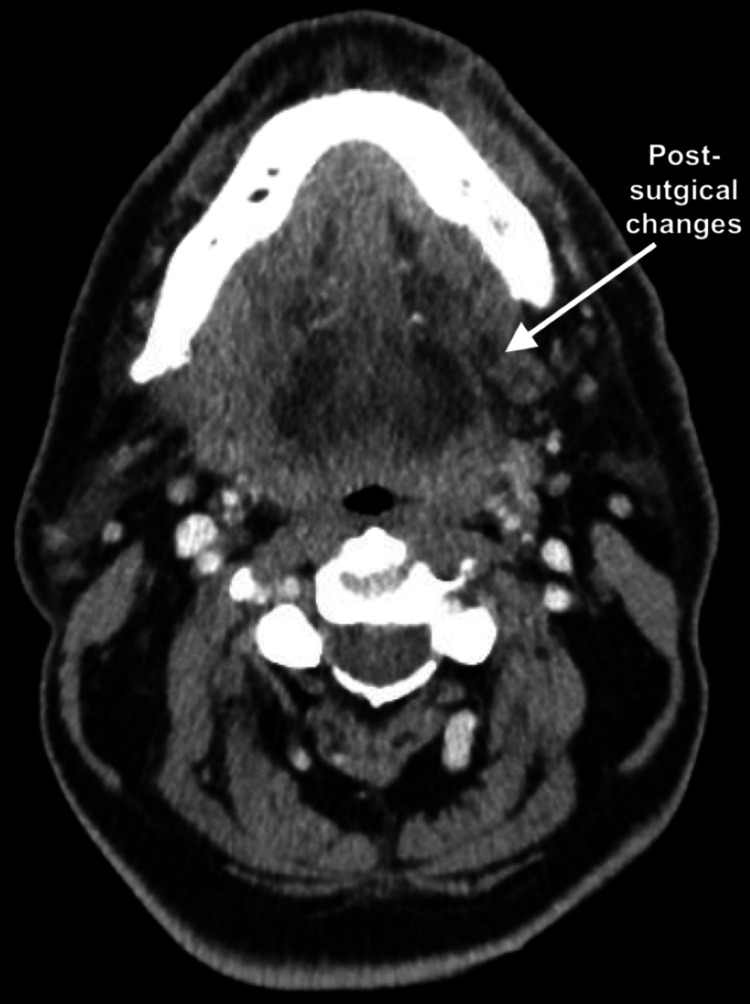
Post-operative day eight CT soft tissue neck Postsurgical changes in submandibular spaces, especially on the left side (white arrow). No evidence of discrete abscess collection

He remained intubated in critical condition, and on postoperative days five and eight, he was again taken back to the operating room for additional debridement. Because of the extensive necrosis and destruction of tissue planes, at no point were the hypoglossal nerves definitively identified. Consequently, the mechanism of injury in this case remains presumptive, based on the anatomic distribution of necrotizing infection and postoperative neurologic findings. Figure 3 depicts the resolved abscess and postsurgical changes in submandibular spaces, most prominently seen on the left, as captured on postoperative day eight.

Furthermore, on postoperative day eight, there was growth of *Candida glabrata* and *Candida albicans* from the neck abscess. The patient underwent superinfection treatment with meropenem and micafungin. The patient’s final course of antibiotics consisted of ceftriaxone and metronidazole for nine days. He remained intubated throughout, and on postoperative day 13, airway evaluation by laryngoscopy demonstrated mild edema that was not concerning and some purulent secretions. The patient's oxygen saturation was >93%, he passed a spontaneous breathing trial, and he remained hemodynamically stable.

Thus, on the morning of postoperative day 14, he was successfully extubated to a high-flow nasal cannula. He was immediately noted to have complete tongue immobility on transoral exam, but was without respiratory distress, stridor, or dyspnea. Laryngoscopy was performed and showed a complete paralysis of the base of the tongue, but the oropharyngeal and hypopharyngeal airway remained patent. Pharyngeal muscles had proper tone and movement. There was also noted to be an excess of secretions. However, given that the patient had a patent airway despite the secretions, as well as adequate oxygenation, the team decided to monitor him closely, and no interventions were pursued. The patient’s subsequent deterioration highlights the difficulty in predicting which individuals with BHNP will progress to acute airway failure. That night, while supine and reportedly asleep, he developed sudden respiratory distress, significant agitation, and a precipitous drop in his oxygen saturations. With persistent desaturations, the patient went into asystole requiring CPR, and after unsuccessful intubation, he underwent bedside cricothyroidotomy. He eventually achieved return of spontaneous circulation and remained without any central neurologic sequela from his respiratory and cardiac arrest. Documentation of the peri-arrest period was limited; however, available charting indicates that the event occurred shortly after supine repositioning, with progressive pooling of secretions and loss of airway patency noted before pulselessness. Additionally, while tongue immobility likely played a central role, other contributing factors to this event may have included postoperative oropharyngeal edema, secretion burden, and the effects of sedative medications. These factors can collectively reduce airway patency. Cricothyroidotomy was revised to a formal tracheostomy that day.

In the coming days following his respiratory arrest, the patient failed a formal swallow evaluation. He remained with complete tongue immobility and was noted to have persistent poor toleration of his secretions with frequent suctioning needs. Observed tracheostomy capping trials while awake led to oxygen desaturation events. Two weeks later, the patient developed a slight bilateral protrusion of the anterior tongue, and repeat laryngoscopy demonstrated mild movement of the tongue base. There was slow but continual improvement in bilateral tongue protrusion, and four months after his hospitalization, he passed a modified barium swallow, as well as a capping trial of his tracheostomy. He was successfully decannulated without any further respiratory events.

## Discussion

BHNP remains a rare clinical entity with variable presentations and outcomes. Review of published cases provides insight into their airway implications and recovery patterns. Other case reports of BHNP have resulted in acute airway decompensation. In some of these cases, the airway decompensation was not immediate but occurred hours to days after extubation. Radcliffe et al. reported a BHNP secondary to harmonic scalpel use, in which the patient required emergent tracheostomy 48 hours after surgery [1]. Rubio-Nazábal et al. reported on a patient who developed BHNP after a prolonged intubation, who failed multiple extubation attempts due to respiratory obstruction and excessive pooling of saliva [2]. After multiple unsuccessful extubation attempts, a tracheostomy was performed. The patient was decannulated after tongue movement had returned and intolerance of secretions had resolved [2]. Altun et al. describe a patient who underwent bilateral stylohyoid ligament resections for Eagle’s syndrome, who was initially stable after extubation, but developed severe inspiratory distress in the post-anesthesia care unit, requiring emergent re-intubation [3]. Following definitive extubation the next day, the patient was noted to have significant dysarthria and dysphagia secondary to BHNP. Her airway was successfully managed in the hospital via continuous use of a nasopharyngeal airway. The nasopharyngeal airway was removed after she began to regain tongue mobility on postoperative day five.

However, not all cases of BHNP result in respiratory distress or dyspnea. Brattou et al. reported a case of BHNP after prolonged intubation that resulted in severe dysarthria, dysphagia, and copious secretions, but no respiratory distress [4]. Mano et al. described a case of atlanto-occipital dislocation in an 11-year-old, resulting in BHNP [5]. The patient likewise had severe dysphagia and a frequent need to swallow to tolerate secretions, but never reportedly developed breathing difficulties.

Regarding our patient, he had complete tongue immobility when first examined after extubation, but did not develop dyspnea or airway compromise until 12 hours later. His respiratory arrest occurred while supine and reportedly asleep, when the pharyngeal tone is at its lowest and the base of tongue is in a vulnerable position to prolapse into the airway. Combined with the inability to properly swallow secretions and clear the obstructing saliva in a timely fashion, this likely contributed to his sudden airway obstruction. However, as this report describes a single patient, the associations observed between positioning, tongue immobility, and acute airway compromise are circumstantial and cannot establish causality.

The tongue plays a crucial role in airway patency. It is mechanically coupled to the pharyngeal and palatal muscles, and broad pharyngeal dilation can be elicited by stimulation of the hypoglossal nerve. This is aptly demonstrated during the use of the Inspire Hypoglossal Nerve Stimulator in patients with obstructive sleep apnea (OSA) [17]. It follows that complete loss of glossal tone can result in reverse phenomena, that is, pharyngeal collapse, oxygen desaturations, and airway compromise. Indeed, this mechanism is what is seen in OSA. While not all cases of BHNP have resulted in dyspnea, enough cases of severe respiratory distress and arrest have occurred so that definitive airway control via tracheostomy should be considered. Nevertheless, the timing and decision to pursue this intervention vary depending on each patient’s clinical course and level of monitoring within the hospital. While early tracheostomy cannot be universally recommended based on a single case, it may be prudent to consider in patients with BHNP who demonstrate progressive dysphagia, secretion burden, or early signs of respiratory instability. Additional recommended strategies to protect the airway include semi-supine positioning of the patient, frequent suctioning, and tracking suctioning requirements. A nasopharyngeal airway can also be considered a tracheostomy when there are signs of poor secretion control and tongue collapse.

A review of the above cases, as well as others describing notable dysphagia, aspiration, and poor secretion management after BHNP, shows recovery of tongue movement ranging from four weeks to eight months. Most cases of BHNP have been secondary to prolonged intubation with neuropraxia secondary to increased cuff pressures; the hypoglossal nerve palsy in all of these cases eventually recovered. In fact, there is only one case that we are aware of in the literature that resulted in permanent bilateral paralysis of the hypoglossal nerve, which was secondary to direct surgical injury [1]. In a review on hypoglossal nerve paralysis, which included both bilateral and unilateral injury, 69.2% (n=45) of cases had complete recovery [16]. Other reports presenting patients who failed to recover include unilateral nerve injury, such as complications to cervical spine surgery, tonsillectomy, and septum correction [18-20]. Thus, patients who undergo a tracheostomy may be reasonably counseled to expect an eventual decannulation, depending on the mechanism of injury. Overall, recommendations from this case and prior reports are necessarily based on observational data, and higher-level evidence is lacking due to the rarity of BHNP.

## Conclusions

BHNP is a rare but potentially life-threatening condition that can lead to profound dysphagia and airway compromise. Although it may develop insidiously, acute decompensation can occur if the airway is not carefully monitored. Our case highlights the importance of early recognition of tongue immobility and vigilance for signs of respiratory distress in patients with recent head and neck infection or surgery. Notably, the optimal exact timing of airway intervention in BHNP has not been explicitly defined in the existing literature, and management decisions must therefore be individualized based on clinical trajectory. Physicians should be aware of the threat of delayed but sudden airway compromise when the uncommon condition of BHNP is encountered, and priority should be given to establishing or maintaining a safe airway until tongue movement recovers to optimize patient outcomes.
